# Genetic structure and trait variation within a maple hybrid zone underscore North China as an overlooked diversity hotspot

**DOI:** 10.1038/s41598-022-17538-9

**Published:** 2022-08-17

**Authors:** Rui Yang, Ya-Wen Deng, Yan Liu, Jing Zhao, Lei Bao, Jian-Ping Ge, Hong-Fang Wang

**Affiliations:** 1 National Forestry and Grassland Administration Key Laboratory for Conservation Ecology in the Northeast Tiger and Leopard National Park, Beijing, 100875 China; 2 Northeast Tiger and Leopard Biodiversity National Observation and Research Station, Beijing, 100875 China; 3 College of Life Sciences, Beijing Normal University, Beijing, 100875 China; 4 Daheishan Administrative District, Beipiao City, 122000 Liaoning Province China

**Keywords:** Ecology, Genetics, Ecology

## Abstract

Tertiary relict flora in East Asia can be divided into northern and southern regions. North China is a diversity hotspot because it can be the secondary contact zone of ancient lineages from the two regions. To test the extent of ancient lineages hybridization and distinguish between the putative species pair *Acer pictum* subsp. *mono* and *Acer truncatum*, we conducted genetic and ecological studies within a maple hybrid zone in North China. Our results suggest that the two lineages of *Acer* coexist in the hybrid zone and that adult and offspring populations show typical bimodal genetic patterns. Hybrid individuals are established at intermediate altitudes between the two parental lineages. Flowering phenology is divergent between lineages, whereas the complex sexual system of *Acer* may ensure pollination among lineages. Leaf and fruit morphologies are different between the northern and southern origin lineages, corresponding to *A. pictum* subsp. *mono* and *A. truncatum*, respectively. Reduced gene flow between lineages suggests that they should be considered as two species. However, large morphological variations within each species and the existence of hybrids offer low reliability of species identification based solely on morphological traits. Our study underscores North China as an overlooked diversity hotspot that requires further study in the future.

## Introduction

**Figure 1:**
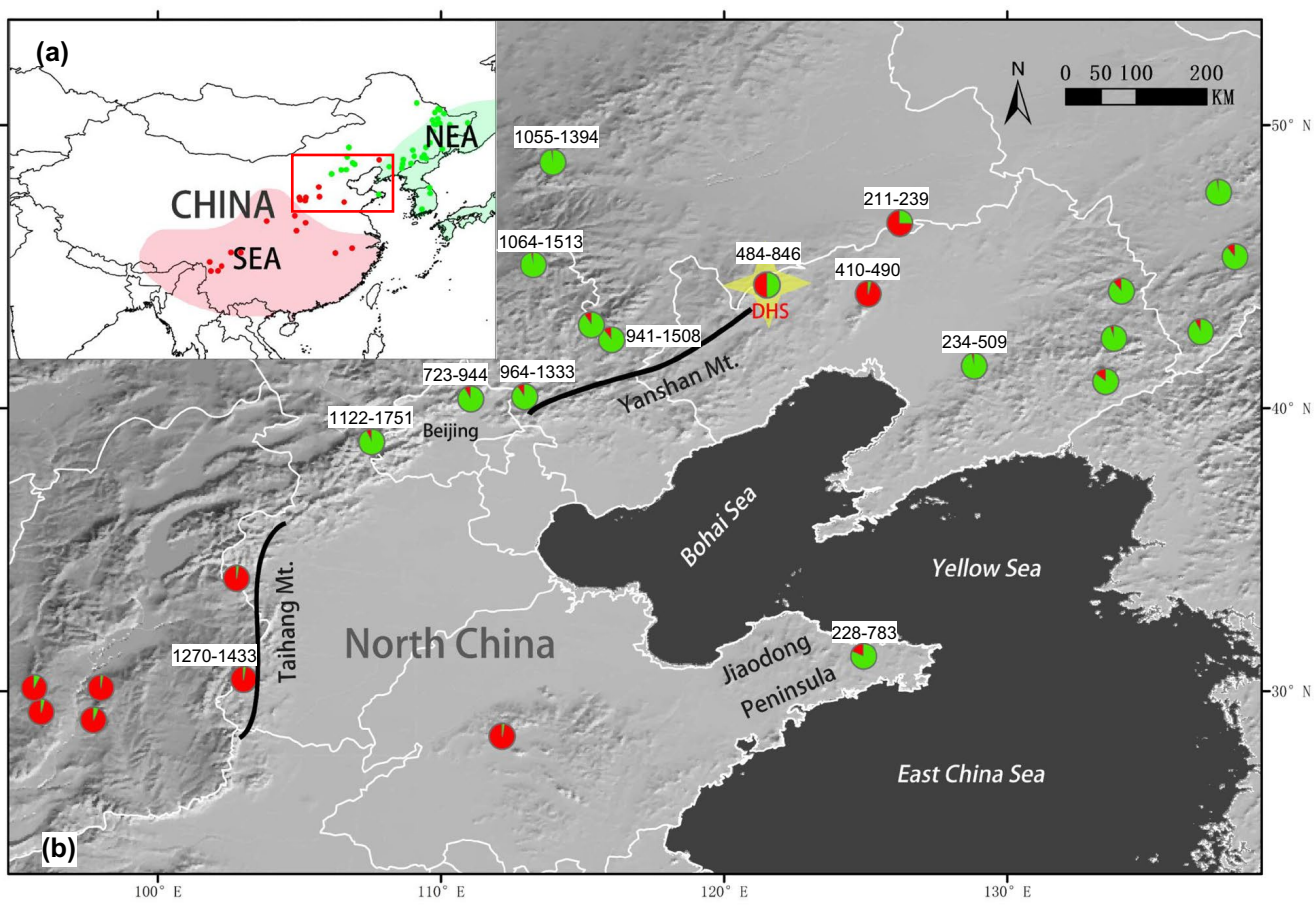
Geographical range of NEA-SEA lineages area and the sampling population locations in North China from GUO et al. 2014. (**a**) Geographical range of two Tertiary relict regions [Southern East Asia (SEA) and Northern East Asia (NEA)] for temperate flora in East Asia, according to Milne and Abbott^5^. The red and green points indicate SEA and NEA *Acer* population locations sampled by Guo et al.^10^. (**b**) An enlarged representation of the area demarcated by the red border in (**a**) showing the geography of North China, which is a key secondary contact zone for tertiary relict NEA and SEA lineages. Colored circles indicate the locations of *Acer* populations. The pie charts of the sampled populations are STRUCTURE results based on a whole-range dataset (see text), with the red and green sectors representing the SEA and NEA lineages, respectively. The numbers adjacent to the circles denote the altitudinal range in meters for all sampled individuals in the population. The focal population in the Daheishan National Nature Reserve examined in this study is indicated by the yellow star. ArcGIS 10.1 software was used for mapping (https://www.esri.com/en-us/arcgis/about-arcgis/overview).

The East Asia region harbors the most diverse temperate flora, where numerous Tertiary relict species have been identified^1–4^. Milne and Abbott^5^ proposed two independent relict species regions within East Asia, one located in South/Southeast China with extensions to the Himalayas (hereafter “SEA”), and the other in a region encompassing Northeast China, the Korean Peninsula, and the Japanese Archipelago (hereafter “NEA”) (Fig. 1a). It is speculated that a climatic barrier demarcated by an arid belt around the North China Plain may mediate the divergence of SEA and NEA flora. SEA–NEA divergence patterns have been reported at intraspecific levels or above^5–7^. Since secondary contact zones between the NEA and SEA lineages have been found at Mt. Taihang and Mt. Yansan, these areas are currently considered major meeting corridors for both lineages^8^ where hybridization may occur. Consistent with the secondary contact zone of ancient lineages, North China, particularly Mt. Taihang and Mt. Yansan, is usually considered a region characterized by intermediate flora composition between tropical, subtropical, and cool temperate regions^9^. Several species have been reported to have divergent lineages or higher genetic diversity populations in this region, including *Juglans spp.*^7^, *Populus spp*.^8^, *Acer spp*^10,11^. *Lindera spp.*^12^, *Betula spp.*^13^, *Prunus spp.*^14^, and *Picea spp*.^15^. Hence, North China can be a diversity hotspot, whereas compared to other temperate flora regions^4^, it is largely overlooked.

*Acer pictum* subsp. *mono* and *A. truncatum* are two closely related species that constitute the predominant components of deciduous forests in North China^16,17^. *Acer truncatum* is mainly distributed in North China and the peripheral area, whereas *A. pictum* subsp. *mono* has a wider distribution from northeast to south China^18–20^. These species have a sympatric distribution in North China. Furthermore, there is wide debate regarding the classification of these two species^21^. Although many scientists treat *A. truncatum* as a separate species^22–25^, some consider it a subspecies of *A. pictum*^26,27^. The two species are generally distinguished by comparing their respective leaf and fruit morphologies. However, given the wide variability in traits, their validity in differentiating between these species is questionable. According to the Flora of China, the leaf base of *A. truncatum* is typically truncated, whereas that of *A. pictum* subsp. *mono* is generally heart-shaped. Furthermore, in *A. truncatum*, the fruit wing is generally as long as the seed, whereas it is 1.5–2 times longer in *A. pictum* subsp. *mono*. However, we observed marked variations in leaf and fruit morphology, even within a single tree (personal observations). Consequently, given the dubious merits of the established morphological diagnoses, it remains unclear whether these are two reproductively compatible species or a single species characterized by large morphological variation.

In a previous phylogeographic study, where both *Acer* species were treated as a species complex, clear NEA-SEA genetic divergence was identified^10^. Furthermore, the authors found that individuals from both lineages were distributed in North China. However, in contrast to other species studied in the same region, only two *Acer* populations were sympatric at a local scale^10^ (Fig. 1b). The Daheishan National Nature Reserve (hereafter “DHS”), located east of Mt. Yanshan, is one of two populations with sympatrically distributed NEA–SEA lineages. Among the 22 sampled individuals in the reserve, 27.3% and 36.4% were NEA and SEA lineages, respectively, with fewer hybrids than expected under random mating^10^. The genetic pattern in the DHS suggests a potential reduction in gene flow between the two lineages. The DHS population provides a unique window to inspect the evolution of SEA and NEA lineages. However, the sample size was very limited in the previous study. Based on more extensive sampling and precise location of each individual in DHS, this study investigated the genetic structure of both adult and offspring populations, monitored flowering phenology, and measured leaf and fruit morphologies. Specifically, we asked the following questions: (1) Do the two lineages show ecological niche differentiation, and are they mixed or isolated on a local spatial scale? (2) Are the two lineages reproductively isolated, and do they differ in terms of flowering phenology? (3) Do the two lineages differ in leaf and fruit morphology, and if so, do these differences correspond to those described for *A. pictum* subsp. *mono* and *A. truncatum* in Floras? Answers to these questions can clarify the evolutionary importance of North China and provide important insights into the evolution of SEA and NEA lineages in the secondary contact zone.

## Result

### Genetic structure of the parental population

**Figure 2:**
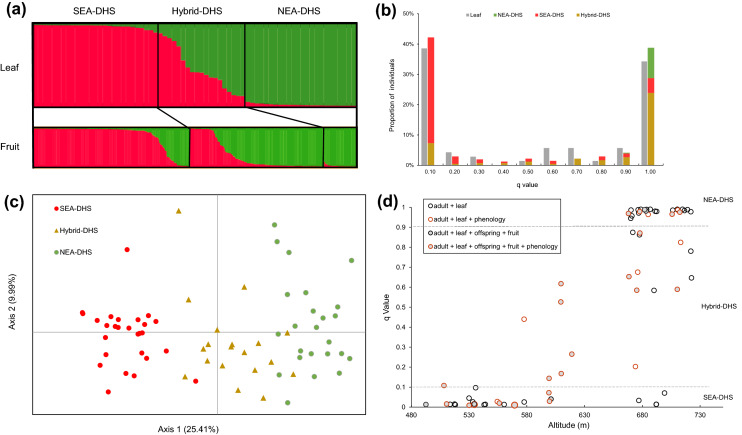
Genetic structure of the parental and offspring population. (**a**) Bar plots illustrating the genetic composition of the adult (leaf) and offspring (fruit) populations in the Daheishan National Nature Reserve (DHS). Each individual is represented by a line partitioned into color segments corresponding to its ancestral proportion. Red color represents the ancestral proportion of Southern East Asia lineage. Green color represents the ancestral proportion of Northern East Asia lineage. Black lines in bar plots of leaf population separate individuals with ancestral proportion (q value) bigger than 0.9 or smaller than 0.1 from hybrids (0.1 < q < 0.9). Black lines in bar plots of fruit population separate individuals from different maternal genetic types. (**b**) Frequency distributions of q value in adult (gray) and offspring (colored) populations. Different colors represent the maternal genetic types of fruits. (**c**) Principal coordinates analysis results obtained for the adult population. (**d**) The q value of 70 *Acer* trees is positively correlated with altitude (Pearson r = 0.83, p = 0.000). Different colors or filled/empty of circles represent individuals used for different analysis in the study. The legend abbreviates adult/offspring genetic structure as adult/offspring, flowering phenology as phenology, leaf/fruit morphology as leaf/fruit. SEA-DHS: Southern East Asia lineage of the *Acer* species complex in the DHS; NEA-DHS: Northern East Asia lineage of the *Acer* species complex in the DHS; Hybrid-DHS: hybrids between SEA-DHS and NEA-DHS lineages.

Based on the lnPD and ΔK values obtained using STRUCTURE, we identified two genetic groups within the DHS *Acer* population (Supplementary Fig. S1). The q value from STRUCTURE analysis represents the proportion of ancestral origin^28^ (Fig. 2a). Among the 70 individual trees, 72.9% were assigned a q value smaller than 0.1 or larger than 0.9, thereby signifying a typical bimodal distribution (Fig. 2b). Individuals with q value greater than 0.9 and consistent genetic origin from the NEA region were defined as the NEA lineage (hereafter “NEA-DHS”), whereas those with values less than 0.1 and with consistent genetic origin from the SEA region were defined as the SEA lineage (hereafter “SEA-DHS”). Individuals with intermediate q value between 0.1 and 0.9 were defined as hybrid genetic types (hereafter “Hybrid-DHS”). Accordingly, we identified 27 SEA-DHS (38.6%), 24 NEA-DHS (34.3%), and 19 Hybrid-DHS (27.1%) (Fig. 2b).

Both principal coordinates analysis (PCoA) and NewHybrids analysis revealed patterns similar to those obtained using the STRUCTURE analysis. The PCoA results indicated that the SEA-DHS and NEA-DHS based on STRUCTURE assignment were divergent and clustered along the first axis of the ordination plot, whereas the Hybrid-DHS individuals were found in an intermediate position of the ordination space between the SEA-DHS and NEA-DHS groups (Fig. 2c).

Newhybrids analysis indicated that 65.7% of individuals were of the parental type (30% SEA-DHS and 35.7% NEA-DHS), and 4.3% were F_2_ hybrids. Among the remaining unclassified 21 individuals, 12 could be classified as F_2_ individuals, and two appeared to be NEA-DHS backcross types with a posterior probability criterion of 0.55.

A comparison of the assignment results obtained based exclusively on the DHS dataset (DHS-only: 70 individuals and 11 loci), and that of the entire species range combined with data from a previous phylogeographic study (whole-range: 1278 individuals and 6 loci, Guo et al.^10^) revealed broadly similar patterns. Based on the whole-range dataset, 27% and 31% of the individuals were SEA-DHS and NEA-DHS, respectively (Supplementary Fig. S2). In this regard, given that for STRUCTURE assignment analysis, a larger number of assessed loci has been reported to be more effective than increasing the number of sampled individuals^29,30^, we have only reported the results obtained based on the DHS-only dataset.

### Genetic analysis of the offspring population

Among the 410 analyzed seeds, 198, 170, and 42 were collected from the SEA-DHS, Hybrid-DHS, and NEA-DHS maternal trees, respectively (Supplementary Table S1). Similar to the parental population, a bimodal pattern was observed in the offspring population (Fig. 2b, Supplementary Table S1). The NEA-DHS maternal trees produced 97.6% pure NEA-DHS seeds, and the remaining seeds (2.4%) were of the backcross type with a high genetic proportion of NEA (q > 0.85). The SEA-DHS maternal trees produced 72.2% pure SEA-DHS seeds, 10.1% pure NEA-DHS seeds, and 17.7% Hybrid-DHS seeds (Fig. 2a). Almost all seeds (q value > 0.5) produced by the SEA-DHS were obtained from a single tree, which was identified as SEA-DHS based on the DHS-only dataset, although it was indicated to be Hybrid-DHS based on the whole-range dataset. The Hybrid-DHS maternal trees produced 17.6% pure SEA-DHS seeds, 57.6% pure NEA-DHS seeds, and 24.7% hybrid seeds.

### Flowering phenology

The sexual system of *Acer* has four phenotypes: duodichogamous, protogynous, protandrous, and male^31^. Hence, there are three functional sex types: (1) “Male I” flowers open earlier than “Female” flowers, with mature stamens, no style, and ovary; (2) “Female” flowers have mature pistils, short filaments, and indehiscence anthers; (3) “Male II” flowers open later than “Female” flowers, with mature stamens, ovaries, and separated stigmas. Duodichogamy is characterized by “Male I,” “Female,” and “Male II” types; protandry by “Male I” and “Female” types; and protogyny by “Female” and “Male II” types^31^.

During the flowering season, we monitored a total of 10,074 flowers produced by 29 trees (Fig. 2d), among which one tree (SEA-DHS) was protandrous, four trees (three Hybrid-DHS and one NEA-DHS) were protogynous, and the remaining 24 trees were duodichogamous. We observed that the blooming phenology of SEA-DHS and NEA-DHS differed significantly to most assessed phenological indices, with a single exception being a marginally significant difference in the peak blooming time of Male I (Table 1). Compared with NEA-DHS, SEA-DHS were characterized by significantly later flowering phenology, with Male I commencement and cessation of blooming being on average two and three days later, respectively. Similarly, the commencement, peak, and cessation of Female occurred later by averages of 4, 4, and 5 days, respectively, whereas those of Male II occurred later by 5, 4, and 5 days, respectively. Furthermore, the duration of blooming was significantly longer in the SEA-DHS group than in the NEA-DHS group by three days. In the case of Hybrid-DHS, the values obtained for all assessed phenological indices were intermediate between those of the two parental types. Among these, the values of the six indices differed significantly from one or the other parental types, with the majority (5/6) differing from those of the SEA-DHS. Thus, phenologically, Hybrid-DHS appeared to be closer to NEA-DHS.

**Table 1:** Flowering phenology of SEA-DHS, Hybrid-DHS, and NEA-DHS.

Statistical index	Genetic type		
	SEA-DHS (N = 9)	Hybrid-DHS (N = 14)	NEA-DHS (N = 6)
Male I commencement	7.44 ± 0.58^a^	6.45 ± 0.49^ab^	5.20 ± 0.86^b^
Male I peak	11.44 ± 1.06^a^*	10.18 ± 0.63^a^	8.80 ± 1.10^a^*
Male I cessation	13.33 ± 1.08^a^	11.09 ± 0.74^ab^	9.8 ± 0.37^b^
Female commencement	14.33 ± 1.08^a^	11.14 ± 0.80^b^	10.00 ± 0.86^b^
Female peak	16.11 ± 1.09^a^	13.29 ± 0.71^b^	11.67 ± 0.67^b^
Female cessation	17.89 ± 1.16^a^	14.57 ± 0.72^b^	12.67 ± 0.84^b^
Male II commencement	18.22 ± 0.66^a^	15.57 ± 0.72^b^	13.67 ± 0.84^b^
Male II peak	19.67 ± 0.62^a^	17.07 ± 0.74^b^	15.50 ± 0.43^b^
Male II cessation	24.22 ± 0.68^a^	22.36 ± 0.77^a^	19.17 ± 0.40^b^
Total duration	18.00 ± 0.33^a^	16.64 ± 0.65^ab^	14.83 ± 0.75^b^

Observations commenced on April 20th. Significant differences between pairs determined at the *p* < 0.05 level based a one-way ANOVA were indicated by different superscript letters. a*: *p* = 0.06.

*SEA-DHS* Southern East Asia lineage of the *Acer* species complex in the Daheishan National Nature Reserve (DHS), *NEA-DHS* Northern East Asia lineage of the *Acer* species complex in the DHS, *Hybrid-DHS* hybrids between *SEA-DHS* and* NEA-DHS* lineages.

However, despite the differing phenology of the SEA-DHS and NEA-DHS, we observed instances of overlap in the blooming periods of male or female flowers in one genetic type with those of flowers of the opposite sex in another genetic type. For example, the peak of Female among NEA-DHS (11.67 ± 0.67) was found to coincide with the peak of Male I (11.44 ± 1.06; *p* = 0.879) in SEA-DHS. Similarly, Female blooming in the SEA-DHS peaked (16.11 ± 1.09) just 1 d after the peak of Male II (15.50 ± 0.43) in the NEA-DHS (*p* = 0.667), which at this time still retained an abundance of male flowers in bloom. In contrast, we detected no overlapping phenology with respect to the blooming of Male I of NEA-DHS or Male II of SEA-DHS with the Female in another genetic type.

### Morphological variation of leaves and fruit

**Figure 3:**
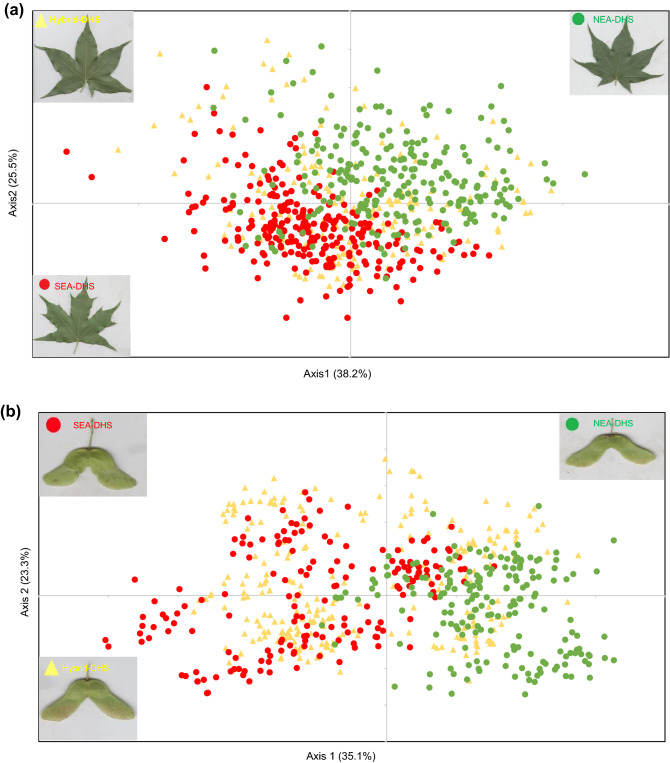
Morphological variation in the leaves (**a**) and fruits (**b**) of southern and northern East Asia lineages of the *Acer* species complex in the Daheishan National Nature Reserve based on principal component analysis. SEA-DHS: Southern East Asia lineage of the *Acer* species complex in the DHS; NEA-DHS: Northern East Asia lineage of the *Acer* species complex in the DHS; Hybrid-DHS: hybrids between SEA-DHS and NEA-DHS lineages.

*Leaves* Among the eight leaf indices, all except InfectionRatio were significantly different between lineages. Generally, the leaves of NEA-DHS were found to have seven lobes, whereas those of SEA-DHS were typically five lobed (Lobes#), thereby contributing to significantly larger leaves in NEA-DHS than in SEA-DHS (TotalArea). Furthermore, NEA-DHS leaves had shorter and wider central lobes (CentralLength and CentralWidth), as well as an earlier and narrower inflection of the central lobes (InflectionLength and InflectionWidth), compared with those of SEA-DHS (Table 2). Six indices had correlation coefficients of less than 0.7, which were used for principal component analysis (PCA) analysis (Supplementary Table S2). The first two axes of the PCA were found to explain 63.7% of the variation in leaf morphology (Fig. 3a), with InflectionLength, CentralLength, and CentralRatio contributing the most to the first axis (38.2%), whereas TotalArea contributed the most to the second axis (25.5%) (Supplementary Table S3). The leaves of SEA-DHS and NEA-DHS plants were largely clustered in separate groups (Fig. 3a). However, all indices were continuous variables with large overlaps between the lineages (Table 2). For example, NEA-DHS had a significantly larger leaf area (21.06–88.70 cm^2^) than SEA-DHS (11.34–70.09 cm^2^). The shape of the central lobe is another major leaf trait that distinguishes between the two species. NEA-DHS had a shorter and wider central lobe (CentralRatio:0.67–2.49), while SEA-DHS had a longer and narrower central lobe (CentralRatio:0.9–3.46).

**Table 2:** Morphological variation in the leaves and fruits of *Acer* trees in the Daheishan National Nature Reserve.

Morphological Indices	Definition	SEA-DHS	Hybrid-DHS	NEA-DHS
	Leaf	N = 269	N = 188	N = 233
**Lobes#**	Number of leaf lobes	5.27 ± 0.04^c^ (5–7)	5.97 ± 0.07^b^ (5–7)	6.59 ± 0.05^a^ (5–7)
**TotalArea**	Leaf area (cm^2^)	32.66 ± 0.66^c^ (11.34–70.09)	38.22 ± 1.04^b^ (18.99–85.52)	46.94 ± 0.85^a^ (11.34–88.70)
**InflectionLength**	Length of inflection point (cm)	1.75 ± 0.04^a^ (0.32–3.59)	1.65 ± 0.06^a^ (0.10–4.09)	1.36 ± 0.06^b^ (0.06–3.91)
InflectionWidth	Width of inflection point (cm)	1.04 ± 0.03^a^ (0.27–2.82)	1.07 ± 0.05^a^ (0.32–3.23)	0.83 ± 0.03^b^ (0.26–3.13)
**InflectionRatio**	InflectionLength:InflectionWidth	1.91 ± 0.04^a^ (0.46–4.11)	1.77 ± 0.05^b^ (0.13–3.54)	1.83 ± 0.04^ab^ (0.06–3.83)
**CentralLength**	Length of central lobe (cm)	4.68 ± 0.05^a^ (2.59–7.45)	4.41 ± 0.07^b^ (2.44–7.65)	4.24 ± 0.05^b^ (2.32–6.25)
CentralWidth	Width of central lobe (cm)	2.46 ± 0.04^c^ (1.24–5.48)	2.66 ± 0.04^b^ (1.57–4.17)	3.03 ± 0.04^a^ (2.07–5.48)
**CentralRatio**	CentralLength:CentralWidth	1.99 ± 0.03^a^ (0.90–3.46)	1.71 ± 0.03^b^ (0.85–2.87)	1.44 ± 0.02^c^ (0.67–2.49)

	Fruit	N = 170	N = 256	N = 189
**FruitAngle**	Fruit opening angle	96.94 ± 1.45^c^ (31.77–149.88)	103.12 ± 1.12^b^ (53.40–157.57)	111.55 ± 1.53^a^ (30.88–157.72)
**JunctionWidth**	Fruit junction width (cm)	0.70 ± 0.01^a^ (0.46–0.99)	0.60 ± 0.01^b^ (0.37–0.83)	0.51 ± 0.00^c^ (0.40–0.65)
**FruitLength**	Length of fruit (cm)	3.85 ± 0.06^b^ (1.93–5.33)	4.17 ± 0.04^a^ (2.40–6.00)	3.53 ± 0.04^c^ (1.52–4.94)
FruitWidth	Width of fruit (cm)	1.87 ± 0.02^a^ (1.26–2.51)	1.80 ± 0.03^a^ (0.87–2.64)	1.29 ± 0.02^b^ (0.76–1.89)
**FruitRatio**	FruitLength:FruitWidth	2.12 ± 0.04^c^ (0.84–3.59)	2.40 ± 0.03^b^ (1.31–3.70)	2.81 ± 0.04^a^ (0.87–3.99)
**SeedLength**	Length of seed (cm)	1.22 ± 0.01^a^ (0.84–1.57)	1.16 ± 0.01^b^ (0.79–1.49)	0.89 ± 0.01^c^ (0.63–1.21)
SeedWidth	Width of seed (cm)	0.79 ± 0.01^a^ (0.54–1.02)	0.73 ± 0.01^b^ (0.49–0.93)	0.61 ± 0.00^c^ (0.43–0.75)
**SeedRatio**	SeedLength:SeedWidth	1.54 ± 0.08^b^ (1.30–1.90)	1.59 ± 0.01^a^ (1.24–2.16)	1.46 ± 0.01^c^ (1.14–1.74)
WingLength	Length of fruit wing (cm)	1.25 ± 0.01^b^ (0.60–1.63)	1.39 ± 0.02^a^ (0.65–2.08)	1.09 ± 0.01^c^ (0.56–1.56)
WingWidth	Width of fruit wing (cm)	0.76 ± 0.01^a^ (0.31–1.13)	0.79 ± 0.01^a^ (0.49–1.21)	0.65 ± 0.01^b^ (0.40–0.92)
**WingRatio**	WingLength:WingWidth	1.70 ± 0.02^b^ (1.04–2.43)	1.78 ± 0.02^a^ (1.22–2.49)	1.68 ± 0.01^b^ (1.00–2.15)
**Wing:Seed**	WingLength:SeedLength	1.06 ± 0.02^c^ (0.57–1.69)	1.20 ± 0.01^b^ (0.79–1.64)	1.24 ± 0.01^a^ (0.79–1.60)

Definitions of morphological indices were presented in Supplementary Fig. S5. The results show the average value ± standard error (minimum–maximum value). Significant differences between pairs determined at the *p* < 0.05 level based a one-way ANOVA are indicated by different superscript letters. Bold indicators were used for subsequent principal component analysis.

*SEA-DHS* Southern East Asia lineage of the *Acer* species complex in the Daheishan National Nature Reserve (DHS), *NEA-DHS* Northern East Asia lineage of the *Acer* species complex in the DHS, *Hybrid-DHS* hybrids between *SEA-DHS* and *NEA-DHS* lineages.

With regard to Hybrid-DHS, the leaves were morphologically intermediate between those of the two parental types (Fig. 3a), as were the values of the assessed morphological trait indices (Table 2).

*Fruits* 11 indices of fruits were significantly different between lineages. NEA-DHS tend to be characterized by smaller fruits (FruitLength and FruitWidth), seeds (SeedLength, SeedWidth and JunctionWidth), and fruit wings (WingLength and WingWidth). Moreover, the seed wings of NEA-DHS fruits are typically oriented at an obtuse angle, whereas those of SEA-DHS fruits tend to be aligned at a right angle (FruitAngle). The length ratio of the wing and seed (Wing:Seed) was larger in NEA-DHS than in SEA-DHS (1.24 vs 1.06, respectively, Table 2). Eight indices had correlation coefficients of less than 0.7, which were retained for PCA analysis (Supplementary Table S4). The first two axes of the PCA explained 58.4% of the variation in fruit morphology (Fig. 3b), with JunctionWidth and SeedLength contributing the most to the first axis (35.1%), whereas SeedRatio and WingRatio contributed the most to the second axis (23.3%) (Supplementary Table S3). The fruits of SEA-DHS and NEA-DHS plants were largely clustered in separate groups, with most fruits of SEA-DHS having negative values in Axis 1, while most fruits of NEA-DHS having positive values (Fig. 3b). Both JunctionWidth and SeedLength in Axis 1 reflect the size of the seed. NEA-DHS had smaller seed (SeedLength: 0.63–1.21 cm, SeedWidth:0.43–0.75 cm), while larger seed in SEA-DHS (SeedLength:0.79–1.49 cm, SeedWidth:0.49–0.93 cm). All indices were continuous variables with large overlaps between the lineages (Table 2).

The morphology of Hybrid-DHS fruits was generally intermediate between that of the two parental types (Fig. 3b), as reflected in the values of the different morphological traits. The exceptions in this regard were FruitLength, WingLength, as well as two ratio indices (SeedRatio and WingRatio), with hybrid trees typically producing longer fruit with longer fruit wings (Table 2).

### Ecological niche divergence between NEA and SEA

We found a positive correlation between q value from Structure analysis and altitude (Pearson’s r = 0.83, *p* < 0.0001) (Fig. 2d). Furthermore, we found significant differences in the altitudinal distributions of the three genotypes. For example, NEA-DHS was primarily distributed at the hilltop of the transect (altitude > 670 m), whereas SEA-DHS was clustered at the foothill (< 600 m), with a few individuals scattered at higher altitudes. The Hybrid-DHS group was found at intermediate altitudes between the parental groups, that is, from the midpoint of the transect to the hilltop (570–730 m).

At the spatial scale of the species range, habitats of NEA and SEA lineages were significantly divergent in 11 out of 19 bioclimatic variables (Supplementary Table S5). Among the 11 temperature-related variables (Bio1–Bio11), only Mean Temperature of Warmest Quarter (Bio10) was not significant between the NEA and SEA habitats. Among the eight precipitation-related variables (Bio12–Bio19), only Precipitation Seasonality (Bio15) was significant between the lineages. Six variables had correlation coefficients of less than 0.7, which were used for PCA analysis (Supplementary Table S5). The first two axes accounted for 82.3% of the variance, with Mean Diurnal Range (Bio2) and Temperature Seasonality (Bio4) contributing the most to the first axis (57.19%), whereas Mean Temperature of Wettest Quarter (Bio8) contributed the most to the second axis (25.11%). The NEA and SEA habitats were mainly divergent along the first axis (Supplementary Fig. S3). Compared with SEA habitats, most NEA habitats were characterized by lower temperatures and larger seasonality of both temperature and precipitation (Supplementary Table S5, Supplementary Fig. S3).

## Discussion

Our results suggest that the NEA and SEA lineages of *Acer* coexist in the DHS region of North China and that both adult and offspring populations show a typical bimodal genetic pattern. We found that each lineage was distinguishable based on its ecological preferences and leaf and fruit morphologies. Furthermore, we found that both lineages hybridize, and hybrid individuals establish at intermediate altitudes between the two parental lineages. These results provide evidence of ecological niche divergence and reduced gene flow between these two lineages.

### Ecological niche divergence has evolved between the NEA and SEA lineages

NEA and SEA lineages in the DHS showed a clear pattern of spatial isolation, with NEA-DHS tending to be predominantly distributed at altitudes greater than 670 m, whereas SEA-DHS were found to be scattered at lower altitudes, with rare overlap between the two lineages. However, despite the limited size of our study population, we believe our findings to be reasonably representative of the regional population, given that we sampled all accessible individuals along a 5-km-long, 20-m-wide transect, with only four or five inaccessible individuals being excluded from sampling. Moreover, consistent with the results of this study, 100 trees sampled during a preliminary survey undertaken in an area near the transect at an altitude of less than 650 m proved to be SEA-DHS individuals (unpublished data). In addition, the trees of both lineages, as well as their hybrids, had a similar age structure, as indicated by diameter at breast height measurements, which for most individuals ranged between 26 and 45 cm (Supplementary Fig. S4), thereby indicating a long persistence of the observed spatial pattern.

Although only one population is understudied, we suggest that spatial isolation along altitudinal gradients may be a common feature of the two species in North China. By checking the altitude of the sampled individuals in the previous phylogeographical study^10^, we found that pure NEA populations found in the north of Mts. Taihang and Yanshan are generally distributed at altitudes higher than 700 m, whereas pure SEA populations in this region are typically found at altitudes lower than 500 m (Fig. 1b), which is consistent with the distribution pattern identified in the DHS.

Our study suggests that the spatial isolation of the NEA and SEA lineages in the DHS mainly reflects temperature-induced niche differentiation. In this study, except for altitude, we did not investigate abiotic or biotic factors along the altitude (such as temperature, soil, light, and other biotic factors), which possibly contribute directly to the spatial isolated pattern of the two lineages and should be involved in future detailed investigations. Based on our indirect evidences, we suggest that temperature is the main factor causing niche differentiation. First, temperature differentiation is the main pattern caused by altitude changes. Second, the main bioclimatic factors between the NEA and SEA habitats investigated throughout the species range were mainly temperature-related variables (Supplementary Table S5). The results of both large and local spatial scales suggest that NEA habitats are usually characterized by lower temperatures than SEA habitats. Hence, since NEA-SEA divergent species usually have sympatric distribution as *Acer* in this study^7,13,32^, it may deserve more studies to test if temperature induce niche divergence in more species in North China.

### Reproductive isolation is developed between NEA-DHS and SEA-DHS lineages

Our results reveal that both adult and offspring populations of the NEA and SEA lineages of *Acer* are characterized by a typical bimodal genetic pattern, with most hybrids being later-generation F_1_ individuals, which indicates a reduction in inter-lineage gene flow. Nevertheless, although the two lineages were isolated in altitudinal distribution, they were still comparatively spatially adjacent (over a range of approx. 6–1800 m). Another study examining the mating patterns and population genetic structure of an *Acer* population in North China^31^ revealed that long-distance pollen dispersal can occur with a high probability. Moreover, it has been reported that the mean pollen dispersal distance of protogynous morphs of *Acer opalus* subsp. *granatense*, is 2.8 km^33^. Hence, spatial isolation is unlikely to be a major factor limiting inter-lineage gene flow. Given the ecological niche divergence in the NEA and SEA lineages, there are opportunities for reproductive barriers to develop. Therefore, the reproductive barrier, rather than the spatial isolation between lineages, is a major factor contributing to the limited inter-lineage gene flow in the DHS.

Based on our observations, the reproductive barrier between the NEA-DHS and SEA-DHS lineages possibly operates before seed development and maturation, which is supported by the consistent genetic structure between the seed and adult populations (Fig. 2b). Generally, prezygotic barriers, such as assortative mating, evolve more rapidly than postzygotic barriers^34^, leading to a pronounced reduction in inter-lineage gene flow^35–37^. Flowering phenology and pollinator community divergence between lineages are common pre-pollination barriers that can contribute to assortative mating^38–40^. This study found that the NEA-DHS and SEA-DHS lineages differed with respect to almost all the flowering phenology indices assessed (Table 1), thereby indicating trait divergence. However, we established that the majority of the individuals surveyed in the DHS were duodichogamous (82.76%) and characterized by two separate functional male stages. Consequently, the prolonged male function can lead to an overlap of the blooming periods of female and male flowers on trees of the opposite lineage (Table 1), even though blooming rhythms differ. Hence, flowering phenology divergence is unlikely to drive the reproductive barrier between lineages of *Acer*. Pollinator community divergence or other post-pollination mechanisms, such as pre-mating barriers associated with the stigma and prezygotic or postzygotic barriers downstream of the stigma^34,41^, can contribute to the reduce gene flow, which still needs further study.

### Morphological divergence of NEA-DHS and SEA-DHS lineages corresponds to A. pictum subsp. mono and A. truncatum

Notably, the NEA-DHS and SEA-DHS lineage trees could be differentiated with respect to the morphologies of both leaves and fruits (Fig. 3, Table 2). The NEA-DHS typically produces larger seven-lobed leaves characterized by a heart-shaped leaf base, whereas the generally smaller leaves of SEA-DHS typically have five lobes and a truncate-shaped leaf base. In fruits, NEA-DHS had smaller seeds and seed wings spreading at larger angles than those produced by SEA-DHS. Furthermore, whereas the wings of SEA-DHS fruits were as long as the seeds (1.06 ± 0.02), those of NEA-DHS fruits tended to be longer than the seeds (1.24 ± 0.01). Although the criteria used to differentiate *A. pictum* subsp. *mono* and *A. truncatum* are not identical among the different Floras^18–20^, our morphological characterization in this study tends to be consistent with the identity of NEA-DHS individuals as *A. pictum* subsp. *mono* and SEA-DHS individuals as *A. truncatum*. Moreover, the clear indication of a reproductive barrier between NEA-DHS and SEA-DHS lineages provides compelling evidence regarding the current classification status of these two controversial species.

Before this study, the morphological comparison between *A. pictum* subsp. *mono* and *A. truncatum* was mainly based on a set of predefined obscure traits^22,23^, which may be the main reason for the inconsistent description between Floras^18–20^. With a clear genetic background, it was possible to define the diagnostic traits of *A. pictum* subsp. *mono* and *A. truncatum.* As shown in our results, the two species were statistically different in terms of many leaf and fruit indices. The shape of the leaf central lobe and seed size were the two major factors contributing to species clustering in PCA analysis (Supplementary Table S3). *A. pictum* subsp. *mono* has a shorter and wider central lobe and smaller seeds, whereas *A. truncatum* has a longer and narrower central lobe and larger seeds. Furthermore, the two species are also statistically different in both flowering phenology and niche. Compared with *A. truncatum*, *A. pictum* subsp. *mono* has an earlier flower phenology though distributed in higher-altitude habitat. However, both species in the DHS were characterized by large-range morphological variation, with a certain degree of overlap between species (Table 2, Fig. 3). Our study involved only morphological variations within a single population, and the extent of trait variation over a larger spatial range is unknown. The morphological overlaps revealed in this sympatrically distributed population still imply low reliability of species identification using only morphological characteristics. Moreover, the presence of hybrids may exacerbate the difficulty in identifying these species based solely on morphology. Thus, species identification based on genetic markers is the most reliable tool, and species-specific markers can be developed based on this study.

## Conclusions

The findings of our genetic structure and trait analyses of *Acer* population in the DHS clarify the identities of *A. pictum* subsp. *mono* and *A. truncatum* as two separate species, which correspond to the NEA and SEA lineages, respectively, identified in a previous phylogeographical study. Within the study region, the two species showed ecological niche divergence along an altitudinal gradient. Interspecific gene flow is uncommon and reproductive barriers before seed maturation have developed. Whereas, reduced gene flow is unlikely resulted by flowering phenology differentiation. The two species are cryptic and have low reliability of identification using only morphological characteristics.

The coexistence of ancient lineages and ecological niche divergence between lineages revealed in this study emphasize the overlooked role of North China as a diversity hotspot. Although *Acer* is the first case reported in North China with clear niche divergence and hybridization between ancient lineages, it should not be the only one. For example, *Juglans mandshurica* (NEA lineage) and *J. cathayensis* (SEA lineage) have been examined in the Mt. Taihang and Mt. Yanshan regions, and a probable hybrid lineage was identified in the latter region^7^. Similar hybridization events between divergent lineages probably occur in North China for another sympatric species, *Betula platyphylla*^13^. *Quercus mongolica* and *Q. liaotungensis* also meet in the north of Mt. Taihang and Mt. Yanshan and extensive hybridization occurred in this region. However, no morphological divergence, or reproductive barriers have been identified in this secondary contact zone^32,42,43^. Furthermore, *Juglans mandshurica* species complex^7^ and *Lindera obtusiloba*^44^, have revealed signals of local adaptation by analysis based on isolation by environment. Hybridization of these pre-adapted lineages in North China can generate high levels of genetic and phenotypic diversity, which is important in the face of climate change and urbanization^45–48^. To date, only a limited number of studies have attempted to explore the NEA and SEA divergence patterns and their secondary contact zone in North China^7,32^. Our study demonstrates hybridization between ancient lineages and the developed reproductive barrier between lineages in North China, which underscore the critical role of North China as a diversity hotspot for temperate flora.

## Materials and methods

### Study area

The DHS is located east of Mt. Yanshan, with altitudes ranging from 590 to 1074 m. The regional climate is a temperate monsoon with an annual average temperature of 4.9–7.5 °C and annual precipitation of 400–450 mm. The vegetation in the reserve is a typical temperate deciduous forest.

### Genetic structure of the parental population

Based on a previous study^10^, we selected a 5.5-km-long, 20-m-wide hillside transect in DHS, where the two genetic types of *Acer* might coexist. Along this transect, from the foot of the hill to the summit, we located all accessible *Acer* adults using the mobile phone GPS toolbox APP, with a location error within 4 m. In total, we located 70 individuals, with an average diameter at breast height of 37.07 ± 1.66 cm (Supplementary Fig. S4). Leaves were collected from all identified individuals and desiccated using silica gel at room temperature until DNA was extracted.

Total genomic DNA was extracted using a Plant Genomic Kit (Tiangen, Beijing, China). Eleven previously used microsatellite loci were used to genotype all the samples (Supplementary Table S6)^10,31^. The 20-µL PCR mixtures contained 10–20 ng DNA, 0.4 µL of each forward and reverse primer (10 µM), 7.7 μL of ddH_2_O, and 10 μL of 2 × TSINGKE Master Mix (Tsingke Biotechnology Co., Ltd.). Reactions were performed in a Veriti™ PCR thermal cycler (Applied Biosystems) using the following amplification program: an initial denaturation at 94 °C for 5 min; six cycles of 94 °C for 50 s, locus-specific annealing temperatures for 50 s, and 72 °C for 30 s; a subsequent 24 cycles of 94 °C for 30 s, locus-specific annealing temperatures for 50 s, and 72 °C for 50 s; and a final extension at 72 °C for 10 min.

Fluorescently labelled PCR products were sequenced using ABI3730XL in a commercial sequencing facility (Tsingke Biotechnology Co., Ltd, Beijing). Allele sizes were determined manually using GeneMarkerv.2.2.0^49^, according to the specific stutter of each locus. All loci were assessed independently by two individuals to reduce scoring errors. Before data analysis, the presence of null alleles and large allele dropout of the 11 loci was checked using Microchecker v2.2.3^50^. Six loci were reported to have null alleles, but no large allele dropouts. Since there were two genetic types in the DHS populations with reduced gene flow between types (see results), it might be no surprise to find null alleles in more than half of the loci^51^. Previous studies have tested the probability of null alleles for the same set of SSR loci in the same species, and no null alleles were found^10,31^. Hence, we retained all 11 loci for subsequent analysis.

To infer the genetic structure of the DHS population, we implemented both a Bayesian clustering approach and PCoA, the latter of which was conducted using GenAlex6.5^52^. Bayesian clustering was conducted using STRUCTUREv.2.3.4^28^ with K values from 1 to 10, an admixture model, and correlated allele frequencies being applied. For each K, we ran 10 independent simulations with a burn-in of 1,000,000 iterations, followed by 2,000,000 rounds of MCMC sampling. The optimal K was determined using Structure Harvester^53^ based on both the maximum likelihood value (lnPD) and the rate of change of lnPD (△K)^54^. To verify the Bayesian clustering results obtained for the 70 trees sampled in the DHS, we conducted an additional STRUCTURE run for K = 2 for a separate dataset comprising the 70 individuals examined in the present study and a further 1208 individuals analyzed by Guo et al.^10^ from sites other than the DHS. Given that Guo et al. assessed only six SSR loci, we used a sub-dataset of 70 individuals screened with the same six loci during the additional calculations, the procedure of which was the same as that described above.

Using NewHybridsv.1.1^55^, we quantitatively determined the posterior probability that each individual fell into one of the following six categories: the two pure parents, first-generation hybrids (F_1_), second-generation hybrids (F_2_), and backcrosses to the two pure parents. The MCMC sampling was set to 100,000, preceded by a burn-in of 100,000 iterations with “Jeffreys-type” priors. Categories were assigned based on the highest probability of greater than 85%.

### Genetic analysis of offspring populations

To determine whether random mating occurred between the different genetic types, we collected seeds from September 2020 and 2021. However, in these two years, we collected sufficient amounts of viable seeds for analysis from only 20 and 17 individuals, respectively (Fig. 2d). Most of the remaining focal trees either failed to produce seeds or only produced a few inaccessible seeds at the canopy. DNA was extracted from 410 seeds that were genotyped using the aforementioned 11 microsatellite loci. Assignment analysis for K = 2, applied using STRUCTUREv.2.3.4, was conducted for the 410 seeds, as well as for the 70 parental trees. The experimental procedure and parameter settings were the same as those described previously.

### Flowering phenology

To investigate the divergence of flowering phenology among different genetic types, we recorded flowering phenology from April 20 to May 18, 2020. Daily observations of flowering were performed for 29 individuals with readily accessed branches, among which there were 9 SEA lineage, 14 hybrid, and 6 NEA lineage trees based on STRUCTURE analysis of the parental population (Fig. 2d). Initially, we selected four branches on each tree that were evenly distributed in the four cardinal directions, which were marked for subsequent daily investigations. The total number of focal flowers produced by each tree was counted at the alabastrum stage, and the sex of each blooming flower was recorded during the monitoring period. The data obtained for the number of flowers of each sex blooming (hereafter Blooming#- “sex”) are presented as numbers recorded daily. No further flowers bloomed on focal branches after May 16.

Based on the daily Blooming#- “sex” data for each tree, we calculated indices of flowering phenology for each individual, including the commencement, peak, and cessation of each sex blooming. Using these data, we examined the potential phenological divergence among genetic types by performing a one-way ANOVA.

### Analysis of leaf and fruit morphology

During August 2020, we collected a total of 690 healthy leaves from 70 focal trees, which were scanned using an HP LaserJet 1100A scanner, and used DIGIMIZERv4.5.2 to measure six indices reflecting leaf size and shape (Supplementary Fig. S5a). Two additional ratio indices were calculated to reflect the central lobe shape (Table 2).

Similarly, we collected and scanned a total of 615 fruits from 22 trees, of which 170 were from SEA-DHS maternal sources, 256 from Hybrid-DHS, and 189 from NEA-DHS, as identified based on STRUCTURE analysis of the parental population (Fig. 2d). Using DIGIMIZERv4.5.2, we obtained the values for eight indices reflecting fruit size and shape (Supplementary Fig. S5b). Four additional ratio indices were calculated to reflect the shapes of the seed, fruit, and wing (Table 2).

All measured indices were tested for genetic types using a one-way ANOVA. To characterize the potential morphological divergence among the different genetic types of leaf and fruit traits, we performed PCA using R 4.1.2. To prevent multicollinearity among the indices, only indices with correlation coefficients less than 0.7 remained for the PCA analysis^56^.

### Ecological niche differences between NEA and SEA

To test potential niche differences between the NEA and SEA lineages, we used the data of 19 bioclimatic variables (1950–2000) obtained from the WorldClim database^57,58^. For each of the 37 NEA and 20 SEA populations (Fig. 1a), based on the six SSR loci in Guo, et al.^10^, the 19 bioclimatic variables were extracted using ArcGIS 10.1 (ESRI). To characterize niche differences among the NEA and SEA populations, PCA analysis was conducted based on bioclimatic variables. In order to prevent multicollinearity, only bioclimatic variables with correlation coefficients less than 0.7 remained for the PCA analysis^56^.

### Statement of ethical approval

All plant material was sampled from natural populations in North China, and no damage was caused to the studied trees. This study was conducted in accordance with local legislation, and permission was issued to collect such samples. Each individual was marked with a tag that could be verified in the future. Voucher specimens were deposited in the Beijing Normal University Herbarium.

## Supplementary Information

Supplementary Information.

## Data Availability

Additional details are provided in Figs. S1, S2, S3, S4 and S5 and Tables S1, S2, S3, S4, S5 and S6. All sample, SSR, flowering, and morphological data are available in the Dryad repository (https://datadryad.org/stash/share/xtIdhSBidbjaHuzda_i7M5Oe1ayytdnZStTy4DXOD98).
