# Supplementary material for: Genetic structure and trait variation within a maple hybrid zone underscore North China as an overlooked diversity hotspot
Source: Sci Rep. 2022 Aug 17;12:13949. doi: 10.1038/s41598-022-17538-9 (PMC9385851; doi:10.1038/s41598-022-17538-9)

**Supplementary information**

**Supplementary Table S1** Bayesian assignment results of 410 seeds from southern and northern lineages *Acer* maternal trees and hybrid maternal trees in the Daheishan National Nature Reserve.

|  | SEA-DHS maternal trees | Hybrid-DHS maternal trees | NEA-DHS maternal trees |
| --- | --- | --- | --- |
|  | N=198 | N=170 | N=42 |
| SEA-DHS seeds | 143 (72.2%) | 30 (17.6%) | 0 (0%) |
| Hybrid-DHS seeds | 35 (17.7%) | 42 (24.7%) | 1 (2.4%) |
| NEA-DHS seeds | 20 (10.1%) | 98 (57.6%) | 41 (97.6%) |

**Supplementary Table S2** Pearson correlation coefficient between six measured morphological indices and two additional ratio indices of *Acer* leaves in the Daheishan National Nature Reserve. Definitions of measured morphological indices were presented in Supplementary Figure S5a. Significant differences between pairs determined at the p < 0.05 level were indicated by *.

|  | **Lobes#** | **TotalArea** | **Inflection Length** | Inflection Width | **Inflection Ratio** | **Central Length** | Central Width |
| --- | --- | --- | --- | --- | --- | --- | --- |
| **Lobes#** |  |  |  |  |  |  |  |
| **TotalArea** | 0.043 |  |  |  |  |  |  |
| **InflectionLength** | -0.113* | 0.292* |  |  |  |  |  |
| InflectionWidth | -0.079* | 0.344* | 0.789* |  |  |  |  |
| **InflectionRatio** | 0.059 | -0.177* | -0.034 | -0.564* |  |  |  |
| **CentralLength** | -0.203* | 0.589* | 0.635* | 0.359* | 0.217* |  |  |
| CentralWidth | -0.136* | 0.718* | 0.097* | 0.148* | -0.179* | 0.292* |  |
| **CentralRatio** | -0.028 | -0.156* | 0.353* | 0.125* | 0.301* | 0.496* | -0.635* |

Bold indices had a correlation coefficient of less than 0.7, and were used for subsequent principal component analysis.

**Supplementary Table S3** Loadings of analysed morphological indices for principal component analysis of *Acer* leaves and fruits in the Daheishan National Nature Reserve. Bold numbers represent the highest contributors.

| **Loadings** | **Axis.1** | | **Axis.2** |
| --- | --- | --- | --- |
| **Leaf** | |  | |
| Lobes# | 0.32 | | 0.29 |
| TotalArea | -0.02 | | **0.76** |
| InflectionLength | **-0.51** | | 0.23 |
| InflectionRatio | -0.20 | | -0.30 |
| CentralLength | **-0.58** | | 0.30 |
| CentralRatio | **-0.51** | | -0.31 |
| **Fruit** |  | |  |
| FruitAngle | 0.32 | | 0.27 |
| JunctionWidth | **-0.49** | | -0.22 |
| FruitLength | -0.15 | | -0.12 |
| FruitRatio | 0.38 | | -0.34 |
| SeedLength | **-0.55** | | 0.14 |
| SeedRatio | -0.18 | | **0.62** |
| WingRatio | 0.16 | | **0.59** |
| Wing:Seed | 0.37 | | 0.03 |

**Supplementary Table S4** Pearson correlation coefficient between eight measured morphological indices and four additional ratio indices of *Acer* fruits in the Daheishan National Nature Reserve. Definitions of measured morphological indices were presented in Figure S5b. Significant differences between pairs determined at the p < 0.05 level were indicated by *.

|  | **Fruit Angle** | **JunctionWidth** | **Fruit Length** | Fruit Width | **Fruit Ratio** | **Seed Length** | Seed Width | **Seed Ratio** | Wing Length | Wing Width | **Wing Ratio** |
| --- | --- | --- | --- | --- | --- | --- | --- | --- | --- | --- | --- |
| **FruitAngle** |  |  |  |  |  |  |  |  |  |  |  |
| **JunctionWidth** | -0.261* |  |  |  |  |  |  |  |  |  |  |
| **FruitLength** | -0.011 | 0.431* |  |  |  |  |  |  |  |  |  |
| FruitWidth | -0.339* | 0.452* | 0.263* |  |  |  |  |  |  |  |  |
| **FruitRatio** | 0.293* | -0.071 | 0.423* | -0.717* |  |  |  |  |  |  |  |
| **SeedLength** | -0.194* | 0.626* | 0.466* | 0.784* | -0.345* |  |  |  |  |  |  |
| SeedWidth | -0.362* | 0.818* | 0.550* | 0.502* | -0.041 | 0.740* |  |  |  |  |  |
| **SeedRatio** | 0.215* | -0.206* | -0.034 | 0.435* | -0.421* | 0.445* | -0.266* |  |  |  |  |
| WingLength | -0.118* | 0.284* | 0.805* | 0.612* | -0.033 | 0.488* | 0.369* | 0.219* |  |  |  |
| WingWidth | -0.457* | 0.603* | 0.733* | 0.437* | 0.081* | 0.530* | 0.707* | -0.162* | 0.635* |  |  |
| **WingRatio** | 0.402* | -0.402* | 0.061 | 0.192* | -0.150* | -0.055 | -0.405* | 0.456* | 0.390* | -0.447* |  |
| **Wing:Seed** | 0.018 | -0.242* | 0.403* | -0.019 | 0.228* | -0.364* | -0.273* | -0.153* | 0.617* | 0.190* | 0.478* |

Bold indices had a correlation coefficient of less than 0.7, and were used for subsequent principal component analysis.

**Supplementary Table S5** One-way ANOVA analysis of 19 bioclimatic variables to reflect the *Acer* habitat divergency of southern and northern East Asia lineages. The results show the average value ± standard error. Significant differences between pairs determined at the p < 0.05 level were indicated by different superscript letters. Six bold bioclimatic variables were used for subsequent principal component analysis. Axi.1 and Axis.2 represented the loadings of principal component analysis, and bold numbers represent the highest contributors.

| Bioclimatic variable | Description | SEA Lineages (N=20) | NEA Lineages (N=37) | Axis.1 | Axis.2 |
| --- | --- | --- | --- | --- | --- |
| **Bio1** | Annual Mean Temperature | **8.36±0.67 ^a^** | **3.43±0.52 ^b^** | **0.446** | 0.389 |
| **Bio2** | Mean Diurnal Range (Mean of monthly (max temp - min temp)) | **9.91±0.3 ^b^** | **11.74±0.22 ^a^** | **-0.478** | -0.117 |
| Bio3 | Isothermality (BIO2/BIO7) (× 100) | **31.55±1.50 ^a^** | **25.11±0.36 ^b^** |  |  |
| **Bio4** | Temperature Seasonality (standard deviation ×100) | **821.65±44.75 ^b^** | **1310.18±28.62 ^a^** | **-0.458** | 0.215 |
| Bio5 | Max Temperature of Warmest Month | **23.24±0.92 ^b^** | **25±0.24 ^a^** |  |  |
| Bio6 | Min Temperature of Coldest Month | **-8.85±1.06 ^a^** | **-21.96±1.03 ^b^** |  |  |
| Bio7 | Temperature Annual Range (BIO5-BIO6) | **32.09±1.33 ^b^** | **46.96±0.99 ^a^** |  |  |
| **Bio8** | Mean Temperature of Wettest Quarter | **17.14±0.76 ^b^** | **18.67±0.29 ^a^** | -0.041 | **0.843** |
| Bio9 | Mean Temperature of Driest Quarter | **-1.86±0.99 ^a^** | **-13.57±0.87 ^b^** |  |  |
| Bio10 | Mean Temperature of Warmest Quarter | 18.18±0.87 ^a^ | 18.7±0.29 ^a^ |  |  |
| Bio11 | Mean Temperature of Coldest Quarter | **-2.25±0.92 ^a^** | **-13.57±0.87** ^b^ |  |  |
| **Bio12** | Annual Precipitation | 834.9±88.67 ^a^ | 714.43±39.34 ^a^ | **0.407** | 0.059 |
| Bio13 | Precipitation of Wettest Month | 171.85±12.74 ^a^ | 180.05±8.28 ^a^ |  |  |
| Bio14 | Precipitation of Driest Month | 10.4±2.80 ^a^ | 6.95±1.28 ^a^ |  |  |
| **Bio15** | Precipitation Seasonality (Coefficient of Variation) | **82.58±4.13** ^b^ | **102.96±1.68 ^a^** | **-0.442** | 0.273 |
| Bio16 | Precipitation of Wettest Quarter | 432.65±31.82 ^a^ | 446.3±20.14 ^a^ |  |  |
| Bio17 | Precipitation of Driest Quarter | 41.05±10.94 ^a^ | 26.03±4.25 ^a^ |  |  |
| Bio18 | Precipitation of Warmest Quarter | 416.3±28.79 ^a^ | 444.24±19.44 ^a^ |  |  |
| Bio19 | Precipitation of Coldest Quarter | 45.35±12.71 ^a^ | 26.03±4.25 ^a^ |  |  |

**Supplementary Table S6** Eleven microsatellite loci used in this study.

| Locus | Repeat Motif | Size Range | Tm (℃) |
| --- | --- | --- | --- |
| Am116 | (CT)_20_ | 210-268 | 56 |
| Am118 | (CT)_16_ | 153-169 | 52 |
| Am340 | (AG)_22_ | 170-202 | 52 |
| Am607 | (AG)_15_ | 126-162 | 56 |
| Am258 | (CT)_17_ | 166-184 | 56 |
| Aca17 | (TC)_17_(AC)_13_ | 390-442 | 54 |
| Aca22 | (AT)_2_(AC)_15_ | 158-174 | 51 |
| Aca24 | (AG)_5_(AC)_6_ | 92-106 | 50 |
| Map09 | (GA)_8_ | 92-116 | 50 |
| AY05 | (CA)_11_ | 200-300 | 61 |
| AY14 | (GT)_8_(GA)_9_ | 163-175 | 65 |


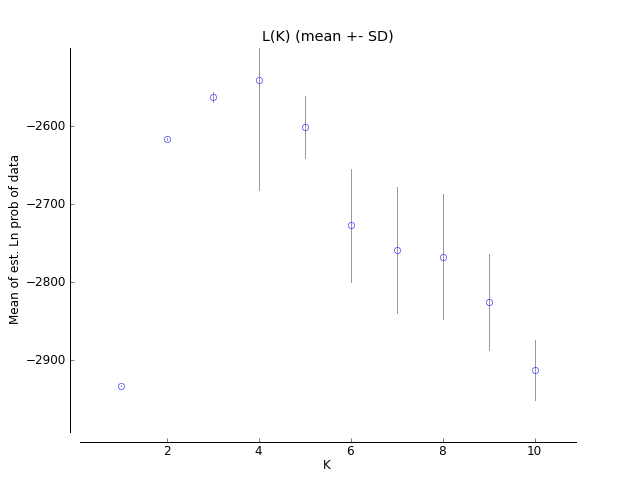

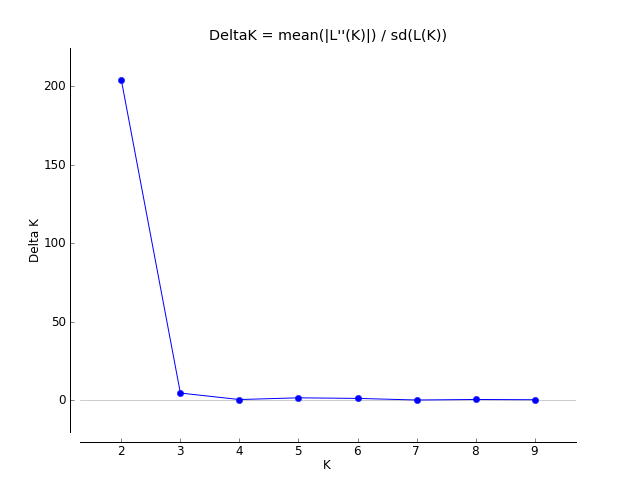


**Supplementary** **Figure S1**. Summary of STRUCTURE results based on DHS-only dataset: lnPD and △K against K.


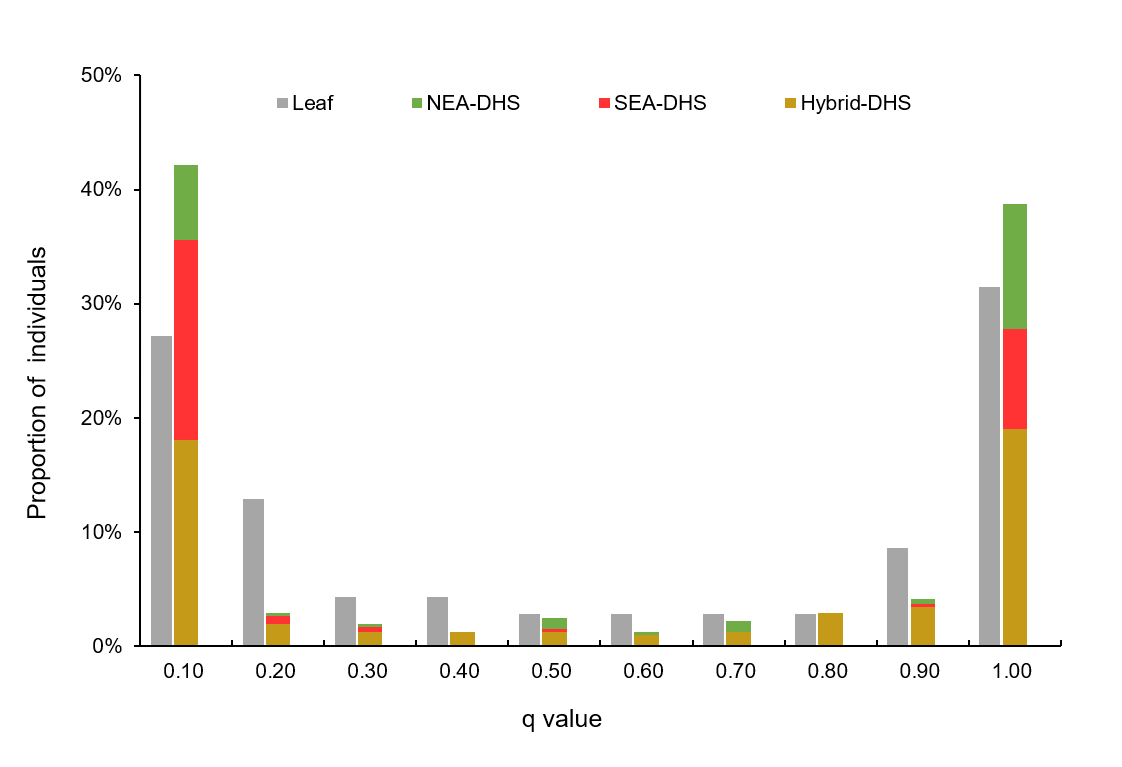


**Supplementary Figure S2**. Frequency distributions of ancestral proportion (q value) of adult (gray) and offspring (colored) populations in the Daheishan National Nature Reserve according to STRUCTURE analysis based on whole-range dataset.
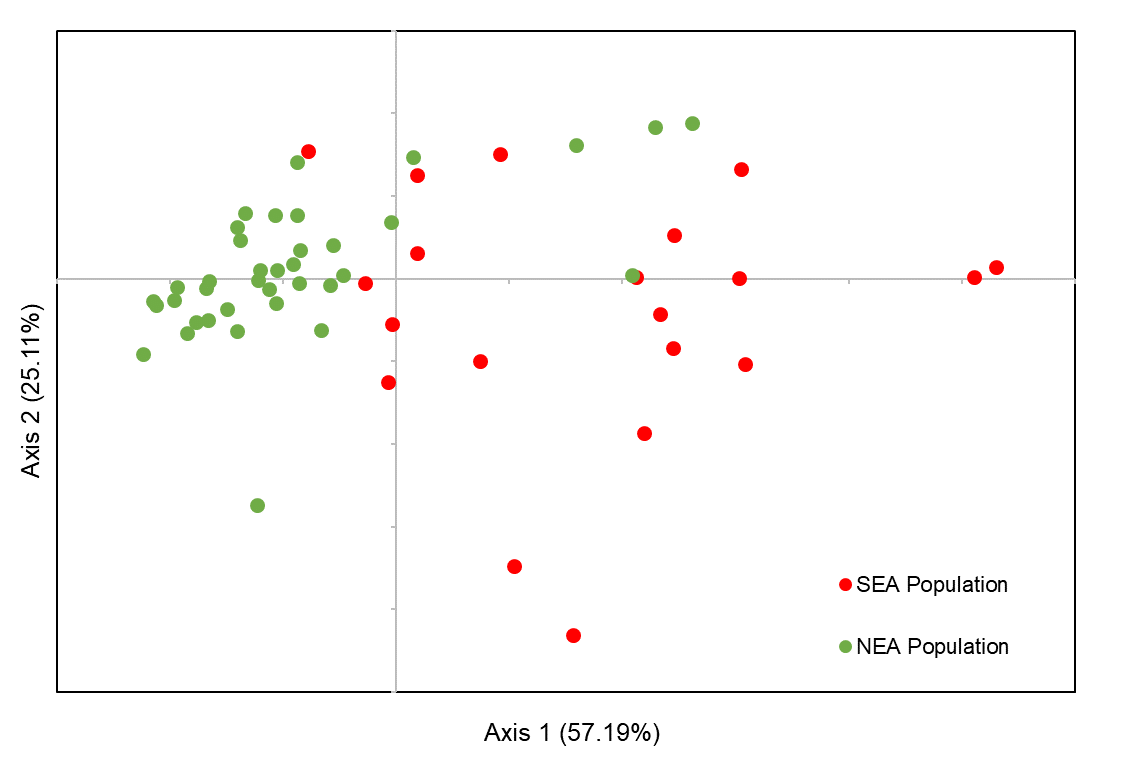


**Supplementary Figure S3**. Habitat variation of southern and northern East Asia lineages of *Acer* species complex based on principal component analysis.

**
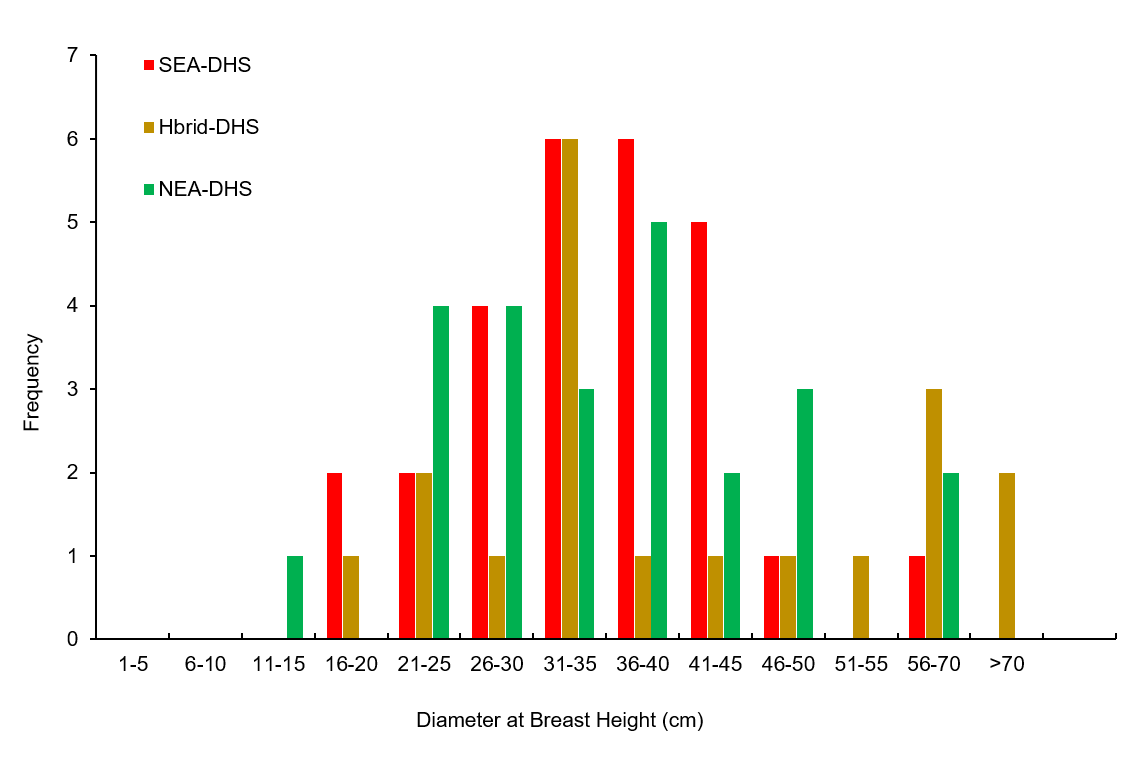
**

**Supplementary Figure S4.** Frequency distribution of Diameters at Breast Height for the 70 sampled trees in the Daheishan National Nature Reserve.

**Supplementary Figure S5.** Definition of morphology indices measure in this study: (a) leaf; (b) fruit.


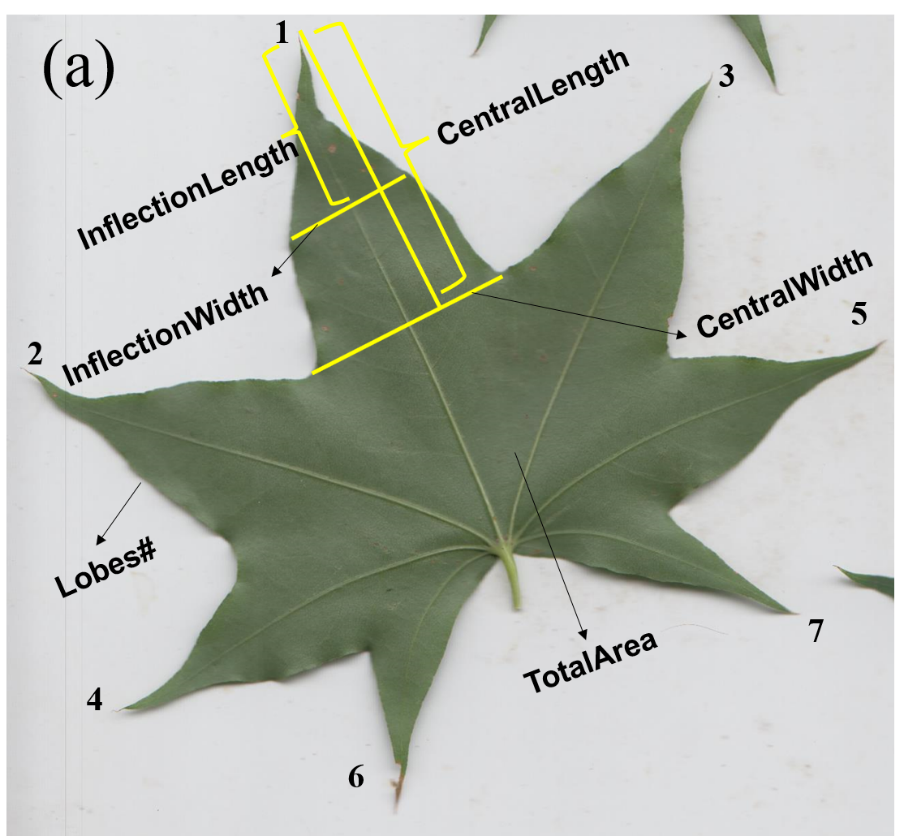

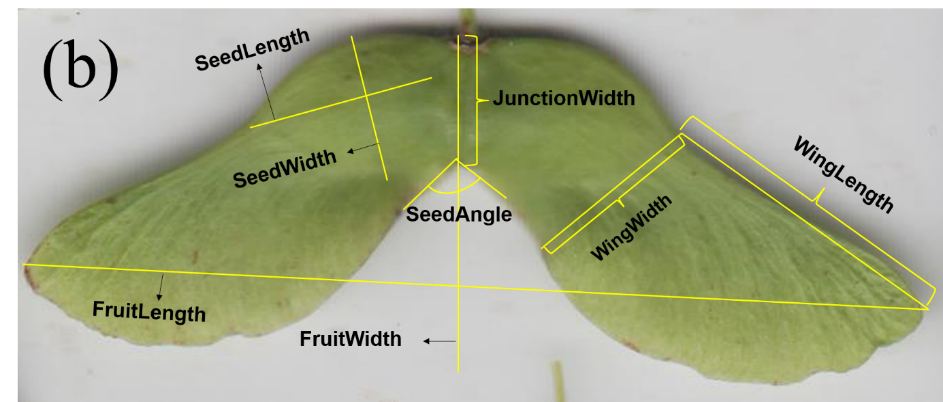

Supplement: Supplementary file 1 — Supplementary Information. [file 41598_2022_17538_MOESM1_ESM.docx]
